# Research on coupling coordination of new quality productive forces and innovation resource allocation based on MLP neural networks

**DOI:** 10.1038/s41598-026-36247-1

**Published:** 2026-01-14

**Authors:** Yanni Liu, Liming Wang, Bian Chen, Haiyang Shan

**Affiliations:** 1 Alibaba Business School, Hangzhou Normal University, Hangzhou, China; 2 School of Management, Hangzhou Dianzi University Information Engineering College, Hangzhou, China; 3 Zhejiang Province Urban New Quality Productive Forces Research Institute, Hangzhou, China; 4 School of Management and E-business, Zhejiang Gongshang University, Hangzhou, China; 5 Zhejiang Huiyi Network Technology Co., Ltd, Hangzhou, China

**Keywords:** New quality productive forces, Innovation resource allocation, Coupling coordination, Dual-tower MLP, Spatial difference, Dynamic evolution, Complex networks, Complex networks, Economics, Economics, Geography, Geography

## Abstract

The synergistic development of new quality productive forces (NQPF) and innovation resource allocation is critical for achieving sustainable and high-quality economic growth. Using provincial data from 2012 to 2022 in China, this study constructs the evaluation framework for NQPF and innovation resource allocation, and employs an unsupervised dual-tower multilayer perceptron (MLP) neural network model to measure the coupling coordination degree. And the spatial differentiation and dynamic evolution of the coordinated degree are further explored. The results demonstrate that the MLP approach offers superior performance in identifying long-term trends while remaining robust to short-term fluctuations. Despite remaining at a primary stage, the overall coordination degree exhibits a distinct upward trajectory. Spatial disparities are primarily driven by interregional differences, with the eastern region exhibiting short-term positive development cycles, the central region showing steady catch-up progress, and the western region facing challenges of marginalization. Moreover, significant spatial spillover effects highlight the influence of geographical proximity, underscoring the importance of cross-regional cooperation and innovation resource sharing.

## Introduction

In the pursuit of global economic competitiveness and sustainable development, nations continually seek novel growth engines. Since 2023, the concept of new quality productive forces (NQPF) has gained prominence in China as a strategic framework aimed at accelerating economic advancement and underpinning sustainability. Distinguished by the innovation-driven nature, NQPF aims to enhance technological innovation mechanisms, optimize the allocation of innovation resources, and foster growth models that integrate efficiency with sustainability^1^. As the strategic significance of NQPF becomes evident, it raises the critical question of how to bridge the gap between NQPF potential and its realization in the real economy? Central to this inquiry is the pivotal role of innovation resource allocation in enabling NQPF development.

As the global economy enters an era of intelligent transformation driven by digitalization, the limitations of traditional productivity have become increasingly apparent, necessitating a fundamental shift toward more advanced and sustainable models. Against this backdrop, NQPF serves not merely the policy slogan but as the comprehensive theoretical framework capturing the essence of innovation-driven development. Theories of productivity have long emphasized its role in shaping societal structures, relationships, and developmental trajectories^2,3^. Classic factors include labor, resources, technology, natural conditions, and social relations, with technological progress increasingly recognized as the core driver of systemic transformation^4^. Building upon this foundation, the national quality policy framework represents an evolutionary advancement beyond earlier stages (industrial productivity, scientific productivity, advanced productivity, and ecological productivity) by integrating high performance, high quality, and low-carbon attributes into a unified growth paradigm^5,6^.

The defining characteristic of NQPF is its reconceptualization of productivity dynamics. It transcends the quantitative expansion and linear efficiency gains typical of traditional productive forces to emphasize qualitative leaps achieved through innovation, digitalization, and sustainable practices^7^. Consequently, NQPF is defined as an advanced form of productive forces catalyzed by scientific and technological innovation, enabling societies to break away from resource-intensive growth models and transition toward a dual pathway of innovation-driven development and resource efficiency. The allocation of innovation resources serves as a pivotal mechanism and metric for the realization of NQPF^8^. By situating NQPF within broader discourses on technological change, sustainable development, and economic transformation, this study treats it as an analytical framework with general relevance for understanding how economies can achieve synergistic coordination between technological progress and resource allocation.

Existing research on innovation resource allocation has explored its connotation, mode, and measurement, but there is a conspicuous gap in quantitative evaluation of the relationship with NQPF^9,10^. First, the connotation of innovation resource allocation is mainly examined from a process perspective emphasizing the efficient integration of scarce resources into innovative capabilities^11^ and an outcome perspective focusing on the structural proportion of innovation factors^12,13^. In terms of allocation mechanisms, the innovation resource allocation mode mainly includes government-led, market-led, and hybrid models, which facilitate feedback optimization through resource absorption, coordination, and recombination, thereby enhancing productivity, technological output, and broader economic development^14,15^. Methodologically, prevailing approaches include indicator-based evaluation systems^16^, measurement of resource distortion degrees^17^, and efficiency analyses^18,19^.

However, the bidirectional coupling between innovation resource allocation and NQPF lacks systematic examination. NQPF drives the efficient and sustainable flow of innovation resources, while high-quality allocation accelerates the development of NQPF. The coordination mechanism between these two systems remains underexplored, particularly regarding quantitative analyses of their synergy and spatial-temporal dynamics^20,21^. Therefore, it is imperative to develop a robust analytical framework to assess their coordination degree, delineate its characteristics, and ultimately uncover the dynamic mechanisms underpinning their coordination development^22^.

**Fig. 1:**
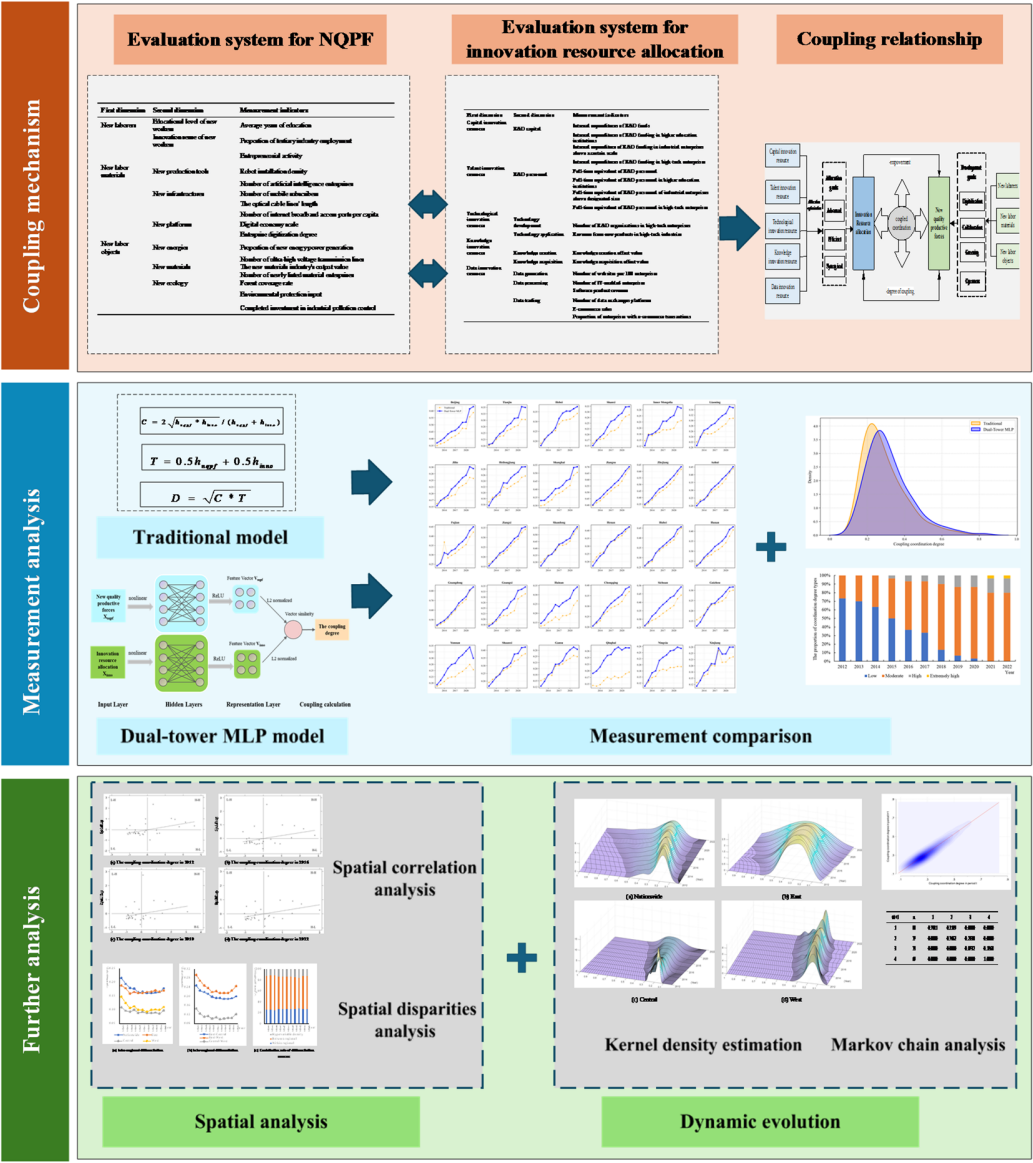
Research flowchart.

To systematically decode the complex relationship between NQPF and innovation resource allocation, this study adopts a progressive analytical framework encompassing nonlinear measurement, spatial decomposition, and dynamic evolution as illustrated in Fig. 1. First, accurate measurement constitutes the prerequisite for analysis. Given the complex nonlinear interactions between NQPF and innovation resources, a dual-tower MLP neural network model is constructed. Unlike traditional linear weighting methods, this deep learning approach captures high-dimensional nonlinear features and synergy premiums within the coupling system to ensure objective evaluation results. Second, decoding spatial patterns is essential for identifying structural barriers. Global and local Moran’s I are utilized to verify the spatial dependence of coordination levels and determine whether high-coordination regions exhibit clustering. Subsequently, the Dagum Gini coefficient is applied to decompose the sources of spatial inequality and pinpoint whether the overall gap stems from intra-regional disparities or inter-regional imbalances. Third, tracing dynamic evolution reveals future trends and path dependence. Kernel density estimation is employed to visualize the distribution shape and polarization trends over time. Furthermore, traditional and spatial Markov chain analyses are integrated to examine state transition probabilities, which is crucial for understanding how geographical proximity influences regional upward mobility and identifying potential spatial poverty traps.

This study offers three primary marginal contributions. First, the coupling coordination degree is measured based on dual-tower multilayer perceptron (MLP) neural network algorithm that captures complex nonlinear interactions between the subsystems of NQPF and innovation resource allocation. Second, the study reveals dynamic evolutionary characteristics and identifies key drivers behind the spatial divergence in the coordination levels. Third, by demonstrating significant spatial spillover effects in regional coordination development, the study proposes policy measures that leverage geographical proximity to foster cross-regional synergy. It is essential to promote the deep integration of NQPF development and the optimization of innovation resource allocation in sustainable development.

## Theoretical analysis and indicator construction of coupling coordination

### Theoretical analysis for coupling coordination relationship

NQPF is intrinsically dependent on the optimized allocation of innovation resources, which serves as a driver for technological progress, human-capital enhancement, high-quality economic development^23^, and the expansion of digital economy^24^. However, research on the sustainable coordination between NQPF and innovation resource allocation remains a critically underexplored topic.

On the one hand, efficient innovation resource allocation acts as a catalytic agent for the advancement of productive forces. It directly fosters technological innovation, thereby elevating production efficiency and output quality^25,26^. Rational financial allocation provides essential material foundation for NQPF, facilitating capital-intensive technological upgrading and R&D activities^27,28^. Furthermore, the agglomeration of high-skilled innovation talents accelerates the iteration and application of production technologies, ultimately promoting economic transformation and high-quality development^29^. As productive forces improve, economic returns can be reinvested in innovation, creating a virtuous cycle for NQPF development. Optimizing innovation resource allocation aims to ensure efficient use of resources. Conversely, innovation resource misallocation has been empirically shown to inhibit the efficiency and quality of productive forces^30,31^. This mechanism transcends traditional growth models by enabling a shift from “quantitative accumulation” to “qualitative leap” through the efficient flow and recombination of innovation resources^32^.

On the other hand, the development of productive forces exerts a strong reverse effect on innovation resource allocation. Regions with more advanced productive forces tend to possess stronger infrastructure and greater R&D capacity, which attract and absorb high-quality innovation resources^33^, leading to a self-reinforcing development pattern. Specifically, the rise of NQPF, characterized by emerging technologies, industries, and business models, reshapes the direction and distribution of innovation resources. For example, the rapid deployment of large language models (LLMs) has spurred investment in data centers and semiconductor development, redirecting capital and talent within the innovation system^34^. Moreover, NQPF drives the spatial reconfiguration of innovation resources. The establishment of high-tech zones and innovation clusters exemplifies how regions guide the concentration and cross-regional flow of resources, breaking traditional geographical constraints and encouraging interregional collaboration. Finally, NQPF promotes evolution in organizational models of innovation, such as innovation consortia and ecosystem-based partnerships. These structures enhance resource sharing, complementarity, and coordination, thereby accelerating technological convergence and system-wide innovation.

**Fig. 2:**
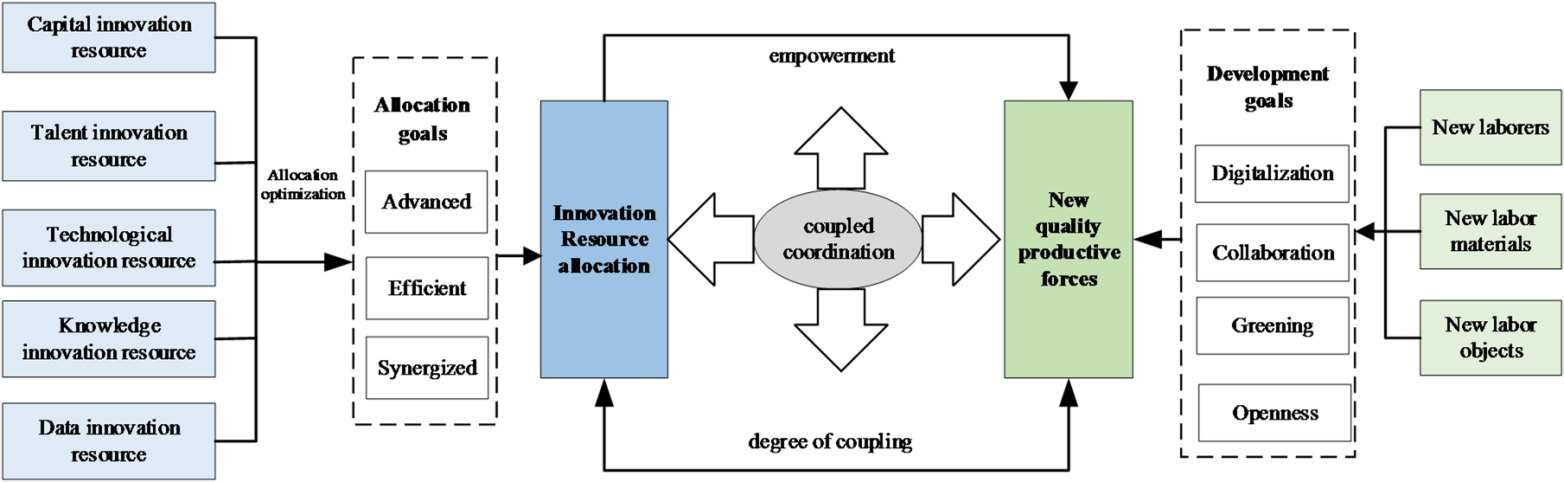
Coupling coordination relationship.

In summary, a bidirectional coupling relationship exists between NQPF and innovation resource allocation where optimized allocation provides the essential inputs for technological and qualitative progress while the development of productive forces guides, attracts, and reorganizes innovation resources. This synergy forms a complex adaptive system whose coordination level determines the sustainability and quality of economic development. This paper evaluates the coordination degree, revealing the current status and evolution trend, and identifying obstacles to coordinated development. The coupling coordination model is illustrated in Fig. 2.

### Indicator system construction

#### Evaluation system for NQPF

Based on the fundamental connotation of NQPF^35,36^, a three-dimensional division framework is used to evaluate the development of NQPF in Table 1. In the construction of the indicator system, we adhered to the principles of scientific rigor and data availability. The system constructs a comprehensive framework that encompasses both the foundational digital capability and the frontier technological applications of NQPF. New laborers represent the qualitative shift from traditional manual labor to high-quality human capital characterized by knowledge intensity, skills, and innovative vitality, serving as the intelligent subject of production. New labor materials embody the technological backbone through the digitization and intelligent upgrading of production tools and infrastructure, acting as the high-efficiency medium that facilitates data flow and automated execution. New labor objects expand the scope of production targets from conventional natural resources to sustainable frontiers, including new energies, advanced materials, and ecological preservation. Collectively, this framework illustrates a systemic mechanism where upgraded laborers utilize intelligent tools to act upon green and high-tech resources, thereby driving the transition toward high-efficiency, high-quality, and sustainable development.

**Table 1:** Indicator system for NQPF.

First dimension	Second dimension	Measurement indicators
New laborers	Educational level of new workers	Average years of education
	Innovation sense of new workers	Proportion of tertiary industry employment
		Entrepreneurial activity
New labor materials	New production tools	Robot installation density
		Number of artificial intelligence enterprises
	New infrastructures	Number of mobile subscribers
		The optical cable lines’ length
		Number of internet broadband access ports per capita
	New platforms	Digital economy scale
		Enterprise digitization degree
New labor objects	New energies	Proportion of new energy power generation
		Number of ultra-high voltage transmission lines
	New materials	The new materials industry’s output value
		Number of newly listed material enterprises
	New ecology	Forest coverage rate
		Environmental protection input
		Completed investment in industrial pollution control

#### Evaluation system for innovation resource allocation

In the study, innovation resource allocation system integrates various innovation-related resources and capabilities, including capital, talent, technology, knowledge, and data. As presented in Table 2, capital serves as the prerequisite and guarantee for innovation activities. Talent serves as the core driver of innovation. Technology lays the groundwork for innovation. Knowledge, measured by knowledge creation and acquisition, provides the foundation for innovation activities. Data, as a strategic emerging resource, is characterized by generation, processing, and transaction.

**Table 2:** Indicator system of innovation resource allocation.

First dimension	Second dimension	Measurement indicators
Capital innovation resource	R&D capital	Internal expenditures of R&D funds
		Internal expenditures of R&D funding in higher education institutions
		Internal expenditure of R&D funding in industrial enterprises above a certain scale
		Internal expenditures of R&D funding in high-tech enterprises
Talent innovation resource	R&D personnel	Full-time equivalent of R&D personnel
		Full-time equivalent of R&D personnel in higher education institutions
		Full-time equivalent of R&D personnel of industrial enterprises above designated size
		Full-time equivalent of R&D personnel in high-tech enterprises
Technological innovation resource	Technology development	Number of R&D organizations in high-tech enterprises
	Technology application	Revenue from new products in high-tech industries
Knowledge innovation resource	Knowledge creation	Knowledge creation effect value
	Knowledge acquisition	Knowledge acquisition effect value
Data innovation resource	Data generation	Number of websites per 100 enterprises
	Data processing	Number of IT-enabled enterprises
		Software product revenue
	Data trading	Number of data exchanges platforms
		E-commerce sales
		Proportion of enterprises with e-commerce transactions

Given integrity and availability of data, this paper selects panel data from 30 Chinese provinces (excluding Tibet, Hong Kong, Macao and Taiwan) spanning 2012 to 2022. Data are derived from *China Statistical Yearbook*,* China Industrial Statistical Yearbook*,* China Science and Technology Statistical Yearbook*,* China Energy Statistical Yearbook*,* Statistical yearbooks of each province*,* China regional innovation capability evaluation report*,* International federation of robotics*,* China Stock Market & Accounting Research Database*,* Enterprise annual reports*,* Tianyancha*,* and the Ministry of industry and information technology*.

This study employs a comprehensive set of computational tools to analyze the coupling coordination. The calculation of the coupling coordination degree based on the dual-tower MLP neural network was conducted using Python 3.9 (https://www.python.org). The Dagum Gini coefficient decomposition, univariate kernel density estimation and Markov chain analysis were performed using MATLAB R2025a (https://www.mathworks.com). Additionally, spatial correlation analysis and joint kernel density estimation were conducted using Stata 18.0 (https://www.stata.com).

## Measuring coupling coordination degree

### Traditional coupling coordination measurement model

The assessment of the interplay between NQPF and innovation resource allocation traditionally relies on the coupling coordination degree model. The entropy weight method is utilized to objectively assign weights based on the dispersion of indicator data, which minimizes subjective bias in calculating the comprehensive development indices for NQPF (${h_{nqpf}}$), and innovation resource allocation (${h_{inno}}$). Subsequently, the coupling degree (*C*) is calculated to quantifies the intensity of the interaction between the two systems:

1 $$C=2\sqrt {{h_{nqpf}}*{h_{inno}}} /({h_{nqpf}}+{h_{inno}})$$

However, the coupling degree solely reflects the strength of interaction and may yield high values even when both systems are at a low level of development. To address this issue of pseudo-coupling, the coordination degree (*T*) is introduced to evaluate the overall development level of the composite system. The relative weights of the two subsystems and are both set to 0.5 to assume equal importance.

2 $$T=0.5{h_{nqpf}}+0.5{h_{inno}}$$

Finally, the coupling coordination degree (*D*) is derived to integrate both the interaction intensity and the development level. This metric provides a holistic evaluation of the synergy between NQPF and innovation resource allocation.

3 $$D=\sqrt {C*T}$$

### Dual-tower MLP measurement model

**Fig. 3:**
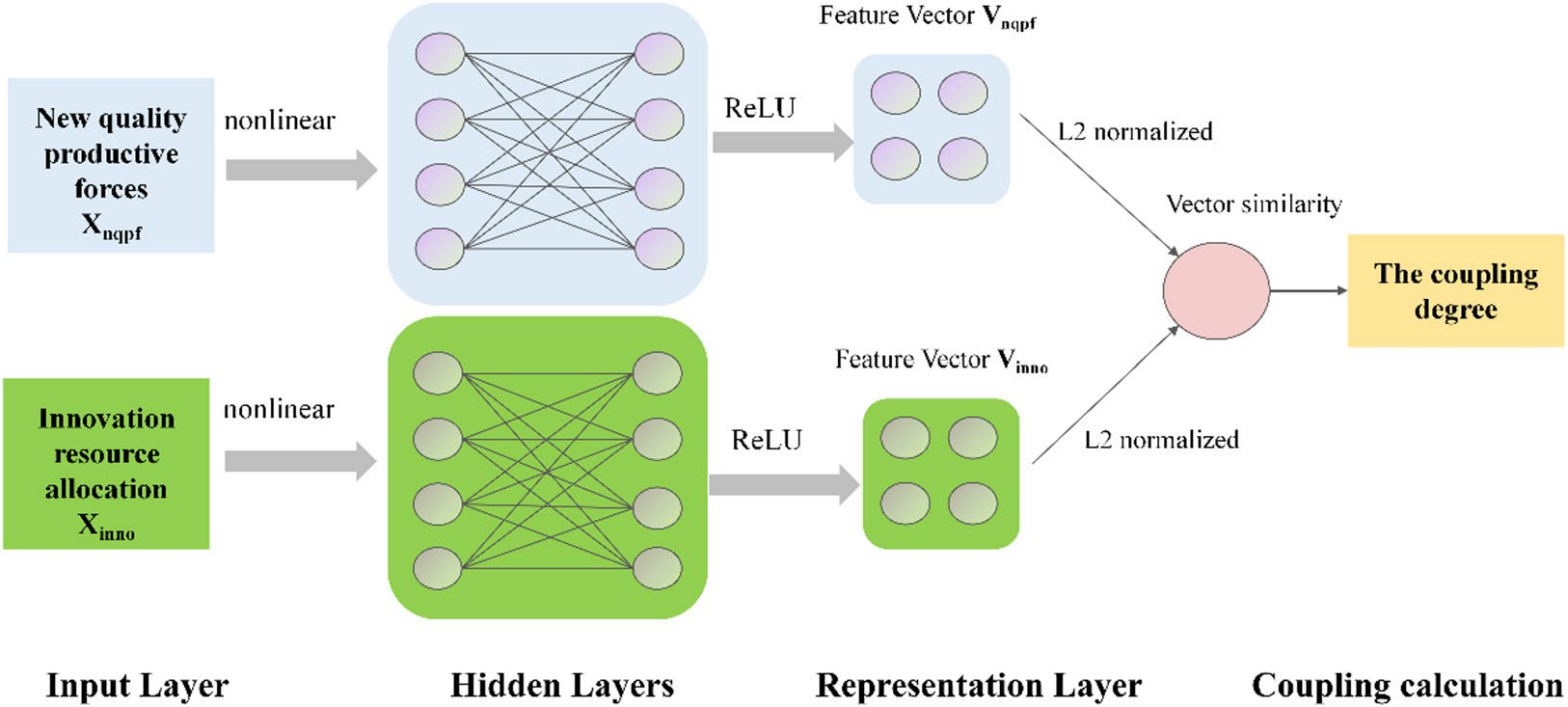
Measurement model based on dual-tower MLP.

While traditional measurement methods offer a basic framework, they rely on linear weighting assumptions that fail to capture the high-dimensional and nonlinear interdependencies between subsystems. To address these limitations and the challenge of lacking ground truth labels for coupling coordination degrees, this study constructs an unsupervised dual-tower deep representation learning model based on the multilayer perceptron (MLP) neural network^37^. Unlike rigid linear models, this approach utilizes a self-supervised mechanism to autonomously uncover the deep structural alignment between NQPF and innovation resource allocation. The specific model architecture is illustrated in Fig. 3.

The model architecture comprises two structurally symmetric yet parametrically independent MLP sub-networks. These two towers respectively receive raw high-dimensional indicator data for NQPF and innovation resource allocation subsystems. Through multiple fully connected layers equipped with ReLU activation functions, the MLP acts as a nonlinear feature extractor that maps heterogeneous raw indicators into abstract high-dimensional latent features. Subsequently, a projection layer compresses the outputs of both towers into a unified common semantic space where they undergo L2 normalization. The final output representation vectors, $\:{V}_{nqpf}$ and $\:{V}_{inno}$ are K-dimensional real vectors with a unit modulus, where K is set to 16 in this study. These vectors function as digital latent representations of the deep logic of the two systems where their directional alignment in vector space signifies the endogenous development characteristics.

To enable label-free training, the study adopts the consistency hypothesis which posits that highly coupled systems should exhibit high directional consistency in their deep representation vectors. Accordingly, the optimization objective is defined by a negative cosine similarity loss function as follows.

4 $$Loss= - \frac{1}{N}{\text{ }}\sum\nolimits_{{i=1}}^{N} {({V_{nqpf,i}} \cdot {V_{inno,i}})}$$

where $\:N$ denotes the sample size, and $( \cdot )$ denotes the vector dot product. Since the vectors are L2 normalized, the dot product equals the cosine similarity, ranging from [-1,1]. To maximize inter-system similarity, the training goal is to minimize this loss function. Consequently, the calculated loss is typically negative (with a theoretical minimum of -1 indicating perfect coupling, and 0 indicating no correlation). During backpropagation, the model continuously adjusts the MLP weights based on this negative gradient, automatically seeking non-linear mapping parameters that minimize the angle between feature vectors, thereby achieving a fully data-driven adaptive coupling measurement.

Upon training convergence, the trained MLP network is utilized to infer the full dataset. The cosine similarity between the output vectors $\:{V}_{nqpf}$ and $\:{V}_{inno}$ is calculated and mapped to the [0,1] interval, defined as the coupling degree ($\:C$). Finally, by combining the coordination degree ($\:T$) calculated, the final modified coupling coordination degree is obtained based on the classic model formula $\:D=\sqrt{C\times\:T}$. This process retains the logical framework of the physical model while incorporating the advantages of MLP neural network in mining non-linear patterns.

### Analysis of measurement results

**Fig. 4:**
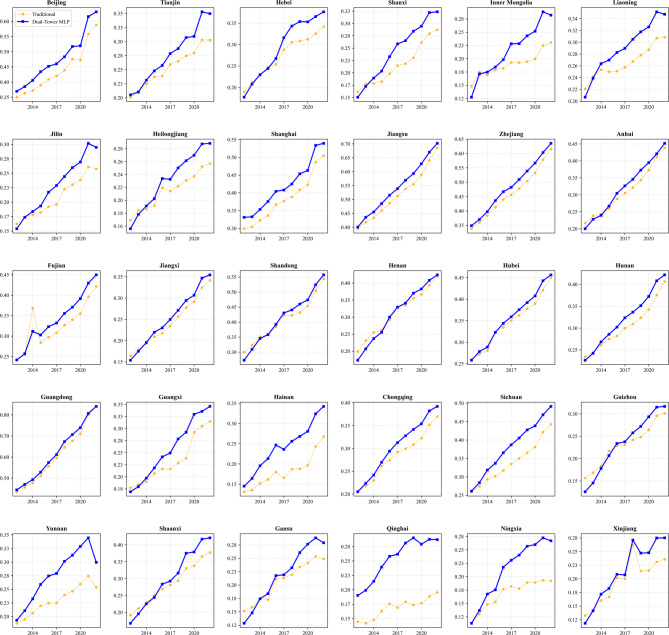
The measurement results.

The comparative measurement results of the coordination degree are illustrated in Fig. 4. It reveals that the traditional measurement method exhibits distinct volatility in specific provinces such as Fujian and Inner Mongolia. These sharp fluctuations likely stem from the sensitivity of the linear weighting mechanism to outliers in single indicators which limits its adaptability to complex dynamic changes. In contrast, the dual-tower MLP model demonstrates superior robustness. It generates smoother trajectories for regions like Beijing and Jiangsu and effectively filters out short-term noise to capture the authentic long-term evolutionary trends of the system.

For regional heterogeneity, the MLP neural network reveals significant divergences from traditional estimates. In eastern developed regions such as Beijing and Shanghai, the coupling coordination degree calculated by the MLP is generally higher than that of the traditional method. This suggests that the traditional linear model underestimates the synergy premiums generated by the high concentration of innovation resources and NQPF. Notably, contrary to the convergence observed in central provinces like Hubei and Hunan, the divergence between the two methods is particularly pronounced in western regions such as Qinghai and Ningxia. In these areas, the MLP model yields significantly higher coordination values than the traditional method. This indicates that the traditional approach may systematically undervalue the development potential of resource-scarce regions, whereas the non-linear capabilities of the neural network can identify latent coordination patterns and region-specific advantages that linear weights ignore.

The traditional coupling coordination degree ($\:{D}_{trad}$) is compared with the proposed deep coupling coordination degree ($\:{D}_{mlp}$) across the full sample. Given the non-normal distribution of the data, the Wilcoxon Signed-Rank Test was employed for non-parametric difference analysis. The results ($z= - 13.709,p<0.001$) indicate a statistically significant difference between the two groups. Notably, the mean of $\:{D}_{mlp}$ is significantly higher than that of $\:{D}_{trad}$, suggesting that traditional linear models may systematically underestimate the synergistic effect between NQPF and innovation resources.

Observing the provincial time-series comparison charts reveals that the divergence between the two models is not uniformly distributed but exhibits distinct structural heterogeneity. This directly validates that the ReLU activation mechanism in the MLP model captures the law of increasing marginal returns within the economic system.

First, scissors difference and synergy premium are shown in developed regions. Taking Beijing, Shanghai, and Guangdong as examples, the results of both models were relatively close around 2014. However, with the passage of time and economic advancement, the growth slope of $\:{D}_{mlp}$ significantly exceeded that of $\:{D}_{trad}$, forming a pronounced expanding “scissors difference”. The primary reason is that traditional models based on linear weighting fail to reflect the “chemical reaction” following factor agglomeration. In contrast, the MLP model captures the network effect when the accumulation of innovation resources exceeds a specific threshold, and their driving force on NQPF exhibits exponential rather than linear growth. The surplus in $\:{D}_{mlp}$ represents the “synergy premium” identified by the MLP model.

Second, low-level overlap and linear applicability is shown in underdeveloped regions. In regions like Qinghai and Gansu, the two curves were highly coincident or intertwined between 2013 and 2016. The main reason is that during the stage of innovation factor scarcity, the interaction between systems manifests primarily as a basic linear input-output relationship, without triggering the non-linear emergence mechanism. The MLP degrades to an approximate linear fit, demonstrating that the model does not generate a systematic “blind overestimation” bias but possesses the capability for adaptive adjustment based on data characteristics.

Third, the MLP model possesses stronger robustness against anomalies in single indicators. For Fujian between 2015 and 2016, the traditional model result exhibited a severe precipitous drop, whereas the MLP result remained relatively smooth despite a slowdown. Retrospective analysis of the raw data reveals that a statistical scope adjustment for a single innovation input indicator caused numerical fluctuations in that year. Traditional entropy weight models are highly sensitive to single indicator weights, leading to distorted measurements. However, the dual-tower MLP structure calculates cosine similarity by extracting high-dimensional feature vectors, focusing on the matching of the holistic feature structure rather than the magnitude of single values. Consequently, the MLP successfully identified that the underlying coupling relationship had not severed, avoiding misjudgments caused by individual data noise.

The divergence between the MLP and traditional models is not a random perturbation. The measurement results indicate that the MLP model successfully overcomes the egalitarian defect of linear models, capturing the non-linear synergistic dividends brought by factor agglomeration in developed regions while maintaining better tolerance for data noise. This distinction underscores the necessity of introducing deep learning methods into the study of NQPF.

**Fig. 5:**
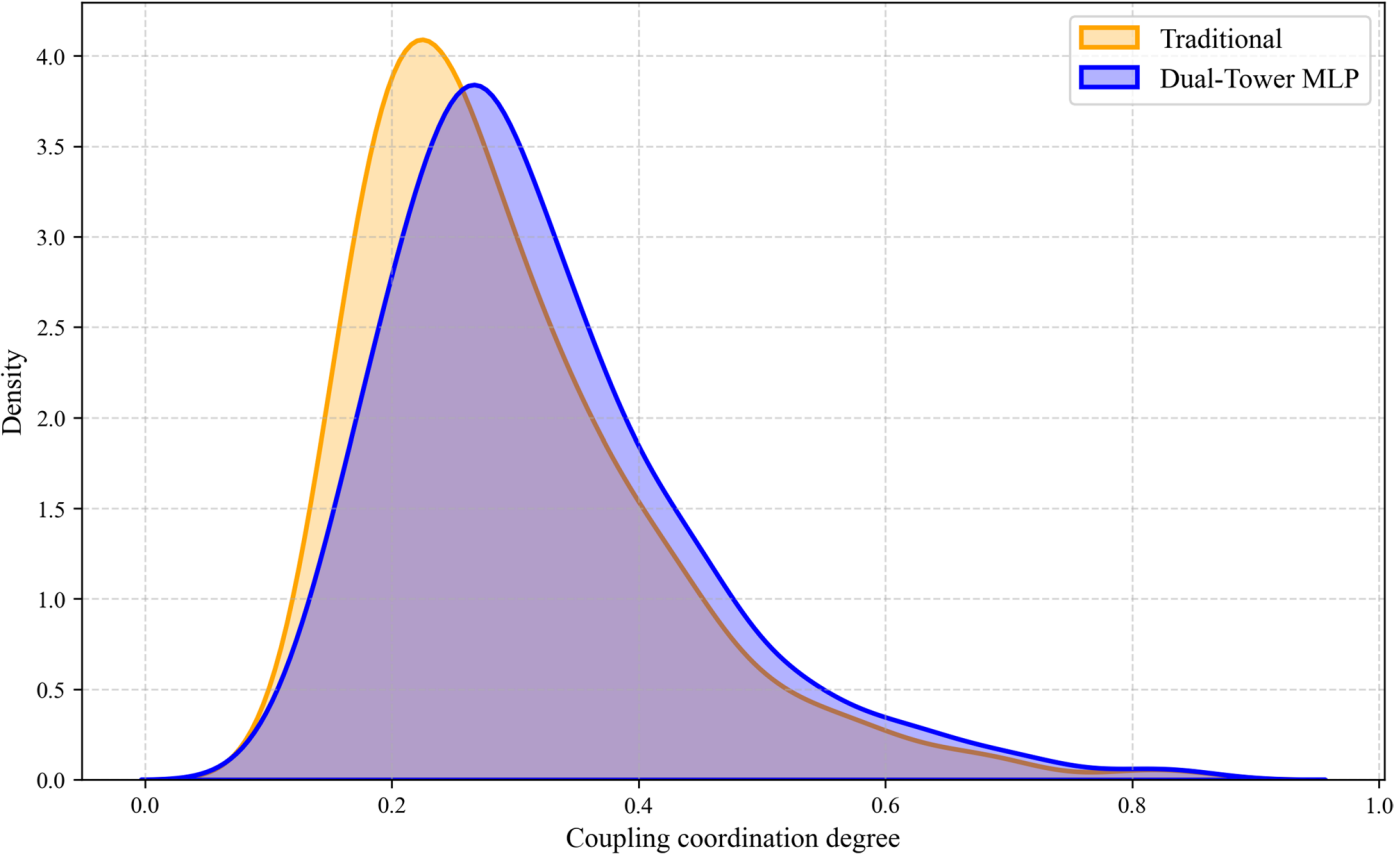
Comparison of measurement results.

The kernel density estimation in Fig. 5 further corroborates these findings. The distribution curve of the dual-tower MLP model exhibits a distinct rightward shift compared to the traditional method which implies an overall higher assessment of coordination levels across the country. Furthermore, the MLP distribution is characterized by a lower peak and a broader span. This morphological difference signifies that the neural network effectively discriminates between regions and reveals a more realistic and differentiated landscape of regional development, whereas the traditional method tends to cluster results in a lower and narrower range. Consequently, the dual-tower MLP model provides a more sensitive and objective instrument for capturing the complex non-linear realities of the coordination between NQPF and innovation resource allocation.

With reference to the existing research and the development of system coordination^38,39^, the coupling coordination degree (*D*) is divided into four types. In the low coordinated coupling stage (*D* $\in$[0, 0.25]), the overall coordinated development has not established a substantial momentum. In the medium coordinated coupling stage (*D* $\in$(0.25, 0.5]), the interaction between subsystems is gradually enhanced. In the high coordinated coupling stage (*D* $\in$(0.5, 0.75]), subsystems interact closely and grow in a highly coordinated way. In the extreme coordinated coupling stage (*D* $\in$(0.75, 1]), the overall coordinated development level is extremely high.

**Fig. 6:**
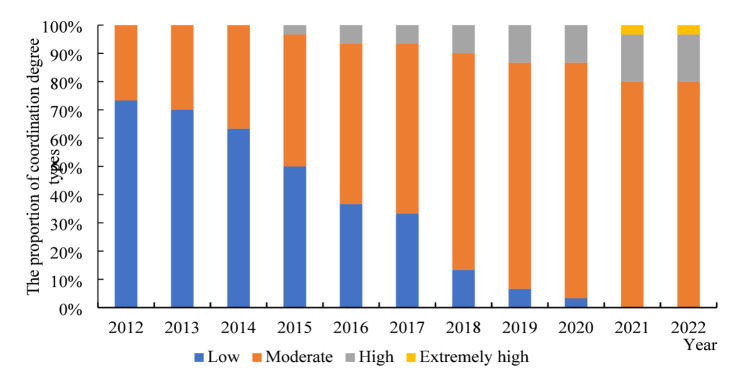
The proportion of coordination degree type.

Based on the measurement results of MLP neural network in Fig. 6, the coupling coordination exhibits the stage-by-stage evolutionary trend. From 2012 to 2015, the low coordination accounted for over 60%, indicating the insufficient motivation for coordinated development and weak interaction between resources. From 2016 to 2019, the proportion of low coordination gradually decreased to 40%-50%, while medium coordination increased and high coordination began to emerge. This reflects that the strengthening interaction between the subsystems, and alignment of innovation resources and NQPF development improves. From 2020 to 2022, the proportion of low coordination continued to decline, medium coordination became an important component, high coordination expanded further, and extreme coordination achieved a notable breakthrough. The overall coordination trend steadily increased over time, suggesting enhanced interaction between NQPF and innovation resource allocation. However, extreme coordination remains at a low level, indicating further optimization of coupling coordination development is needed. Continued efforts to enhance the adaptation of resource allocation and improve the systematic linkage mechanism are essential to promote coordination development.

## Spatial analysis of coupling coordination

Given the vast disparities in resource endowments and technological foundations across China, the coordinated development of NQPF and innovation resources is inherently uneven and spatially stratified. Therefore, relying solely on temporal trends is insufficient to capture the full picture of regional development. This section systematically investigates the geographical dimension to uncover how the coordination degree is spatially distributed and whether it exhibits specific agglomeration patterns or core-periphery structures.

### Spatial correlation analysis

According to the first law of geography, regional economic systems are functionally interconnected rather than isolated islands. Before analyzing specific disparities, it is methodologically requisite to verify the existence of spatial dependence among provinces. This analysis employs spatial statistics to test whether the development of one region significantly influences its neighbors, thereby providing the empirical premise for considering spatial spillover effects in policy formulation.

As a correlation coefficient for characterizing spatial relationships, global and local Moran’s I are analyzed to examine the spatial correlation of the coupling coordination degree. A spatial economic-geographical weight matrix is constructed by integrating geographical distance and economic development. Specifically, the spherical distance between regions is calculated based on latitude and longitude, while economic development is measured by per capita GDP. The weight matrix ${W_{ij}}$ reflects that regions with closer geographical distance and similar economic development may exhibit stronger interactions. The specific construction method is as follows:

5 $$\:{W}_{ij}={W}_{dist}\times\:{W}_{econ}=\left\{\begin{array}{cc}\frac{1}{{d}_{ij}^{2}}\times\:\frac{1}{\mid\:{\overline{PGDP}_{i}}-{\overline{PGDP}_{j}}\mid\:}&\:i\ne\:j\\\:0&\:i=j\end{array}\right.$$

where $\:{d}_{ij}$ denotes the spherical distance between the administrative centers of province$\:\:i$ and province $\:j$, calculated based on latitude and longitude coordinates. The inverse squared distance ($\:\frac{1}{{d}_{ij}^{2}}$) reflects the attenuation of spatial spillovers with geographical separation. $\:{\overline{PGDP}}_{i}$ represents the average per capita GDP of province $\:i$ based on long-term averages. The term ($\:\frac{1}{\mid\:{\overline{PGDP}_{i}}-{\overline{PGDP}_{j}}\mid\:}$) signifies the economic distance. A smaller economic gap yields the larger weight, implying that regions with similar economic structures have stronger absorptive capacities and interaction intensities for NQPF.

#### Global Spatial autocorrelation

To capture the macroscopic characteristic of the spatial footprint, this subsection utilizes Global Moran’s I to quantify the overall intensity of spatial clustering across the entire study period. It serves to determine whether the coordinated development follows a statistically significant pattern of spatial agglomeration on a national scale, confirming whether high-performance regions tend to geographically converge.

As presented in Table 3, the global Moran’s I index remains positive and statistically significant at the 5% level throughout the study period. This statistical evidence confirms that the coupling coordination degree between NQPF and innovation resource allocation is not spatially random but exhibits a strong positive spatial correlation. Specifically, regions with similar coordination levels tend to cluster geographically, meaning that high-coordination provinces are typically adjacent to other high-coordination provinces while low-coordination regions are clustered together. This phenomenon highlights that the development of region is closely linked to and influenced by its neighbors through spatial spillover effects.

**Table 3:** Global moran’s I.

Year	2012	2013	2014	2015	2016	2017	2018	2019	2020	2021	2022
Moran’s I	0.287	0.264	0.302	0.250	0.215	0.193	0.182	0.175	0.188	0.213	0.214
*P*	0.005	0.007	0.003	0.010	0.020	0.031	0.036	0.041	0.032	0.020	0.020

For the trend changes, the global Moran’s I exhibits the “rise-fall-rise” pattern. The index initially climbed to a peak of 0.302 in 2014 which indicates a strengthening of spatial agglomeration during this early phase. Subsequently, the index experienced a gradual decline and reached a trough of 0.175 in 2019. This downward trend suggests a temporary weakening of spatial dependence, possibly due to the localized rapid growth of certain regions that widened the gap with their neighbors. However, from 2019 to 2022, the index rebounded to 0.214. This resurgence indicates a re-strengthening of spatial correlation and suggests that regional integration strategies and cross-regional resource allocation policies have begun to take effect. These fluctuations underscore the dynamic nature of spatial interactions and highlight the necessity of adopting differentiated regional development strategies that account for these evolving spatial patterns.

#### Local spatial autocorrelation

Since global indices may mask localized heterogeneities and outliers, it is crucial to pinpoint specific spatial anomalies within the broader network. By utilizing local Moran’s I, this analysis visualizes the exact locations of “High-High” synergy clusters and “Low-Low” depression areas. This identification of growth poles and collapse basins is essential for implementing differentiated regional intervention strategies.

The spatial agglomeration characteristics is analyzed by Moran scatter plot including four quadrants, such as H-H (High-High) promotion aera, L-H (Low-High) transition area, L-L (Low-Low) backward area, and H-L (High-Low) radiation area. Most scattered points fall into the H-H and L-L quadrants, indicating the formation of a spatial pattern dominated by homogeneity and supplemented by heterogeneity. The homogeneity is stronger than the heterogeneity, suggesting an evident spatial club convergence phenomenon.

**Fig. 7:**
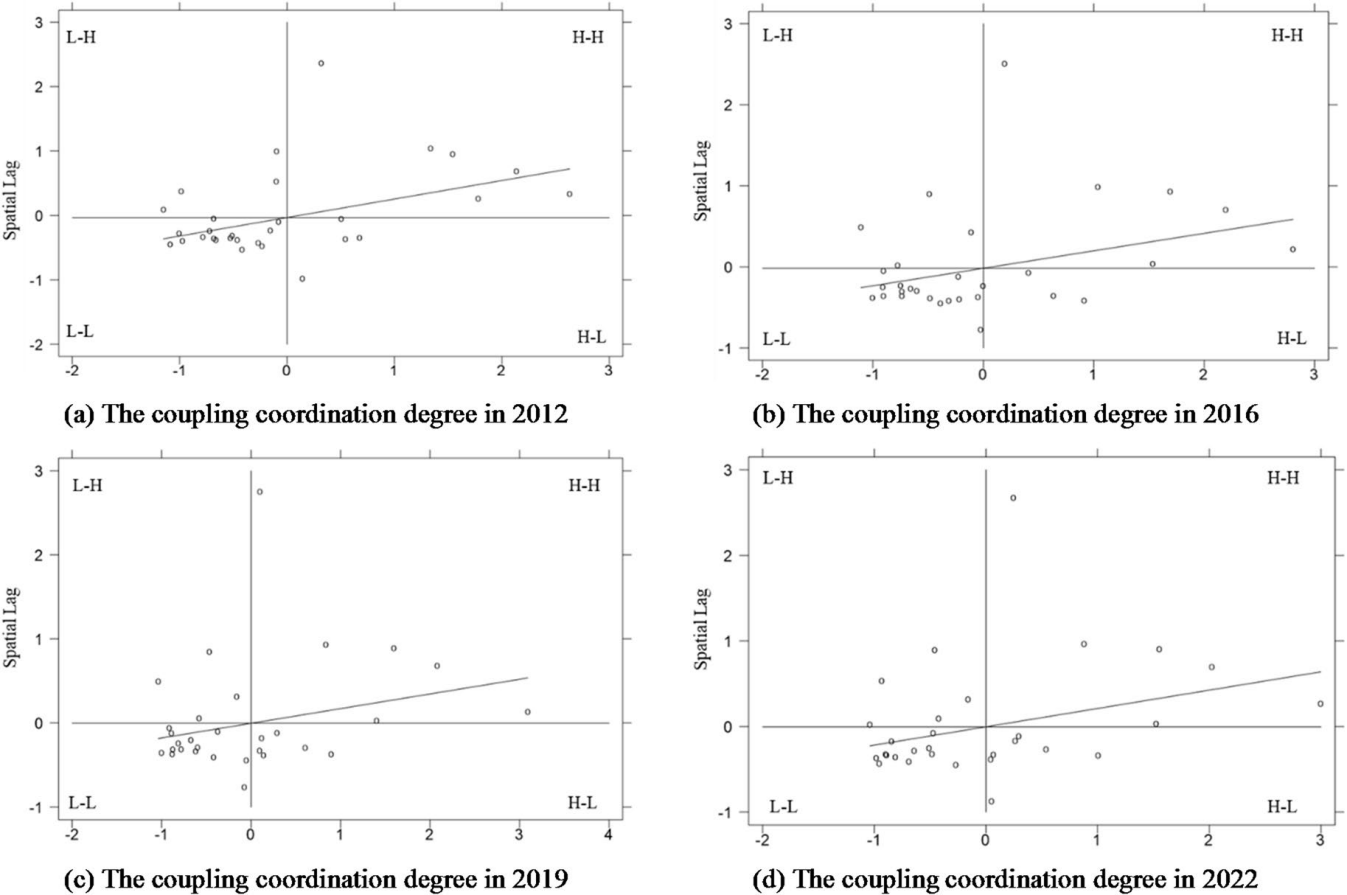
Local Moran scatter plot.

According to Fig. 7, central and western inland regions are primarily concentrated in H-H promotion areas, whereas majority of central and western inland regions are located in L-L backward areas. The eastern coastal areas with superior conditions, exhibit more pronounced spatial spillover effects on coordination degree. For example, five provinces including Beijing, Shanghai, Jiangsu, Zhejiang, and Guangdong consistently fall within in the H-H promotion area, enhancing the coordination degree of neighboring provinces through their radiation effects. Provinces in the central and western regions such as Gansu, Qinghai, and Xinjiang have always been in the L-L backward areas, which are the lowlands of the coordination degree and should be prioritized for future improvements. These provinces with relatively disadvantaged development endowments, continuously stay in the backward areas and fall into the vicious cycle of “the strong get stronger while the weak get weaker”.

Additionally, the number of provinces in the H-H type and L-L type has changed from 6 to 18 in 2012 to 6 and 12 in 2022 respectively. The radiative effect of high-coordination provinces on the surrounding backward provinces outweighs their polarization effect. The H-L radiation areas and L-H transition areas have become the fault zones for the radiation effect. For example, Shandong remained in the H-L type throughout the investigation period. Under the echo effect, this agglomeration area is still in a polarization development stage, attracting production resources from neighboring provinces and squeezing the improvement space for coordination degree in surrounding less developed provinces. This leads to a development deficit in the L-H type involving some neighboring provinces.

### Spatial disparities analysis

**Fig. 8:**
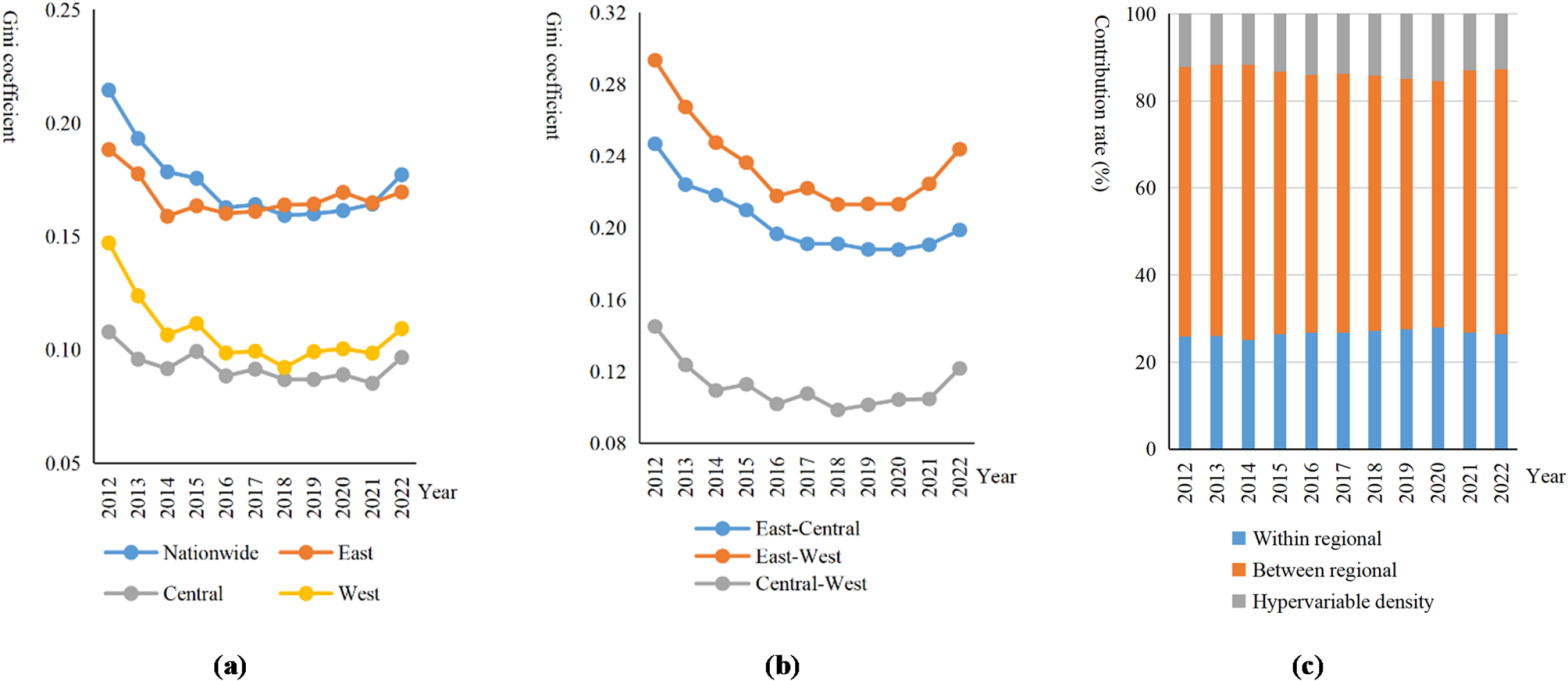
Gini coefficient decomposition of coordinated development degree. (**a**) Intra-regional differentiation, (**b**) inter-regional differentiation, (**c**) contribution rate of differentiation sources.

Merely identifying spatial unevenness is insufficient, making the rigorous quantification of the magnitude and distinct sources of inequality vital for targeted policymaking. Previous research has identified regional variations in the coupling coordination, but the underlying reasons for these differences remain unclear. The Dagum Gini coefficient method can quantify spatial disparities and elucidate the intrinsic causes. This decomposition allows us to trace the root causes of regional stratification and determine whether inequality stems primarily from local imbalances or cross-regional structural gaps. In Fig. 8, this paper measures the overall Gini coefficient and its decomposition coefficients for the coupled coordinated degree.

The overall Gini coefficient of the coupled coordinated development exhibits a “decline-stabilization-rebound” trajectory (U-shaped trend). As shown in Fig. 8a, the nationwide Gini coefficient dropped significantly from 2012 to 2016, indicating that regional disparities initially narrowed, likely due to effective national balancing policies. However, a slight rebound has emerged since 2020. The eastern region maintains the highest level of internal imbalance throughout the period. This is likely due to the polarization effect where innovation resources are highly concentrated in core metropolises, widening the gap with peripheral cities within the region. Conversely, the central and western regions show relatively lower internal Gini coefficients, which also followed a downward trend before stabilizing, contradicting the notion of a continuous marked increase.

Regarding inter-regional differences in Fig. 8b, the disparity between the eastern and western regions remains the largest contributor to the overall gap. The trend mirrors the nationwide pattern: a sharp convergence between 2012 and 2016, followed by a renewed divergence after 2019. This suggests that while early strategies like the western development drive were effective, the recent emergence of NQPF —which rely heavily on high-end innovation factors—may be re-concentrating in the east, causing the gap to widen again. The difference between the central and western regions remains the smallest and most stable, with only a mild uptick in recent years. The eastern region should leverage its radiation effect to support other regions, while the central and western regions require targeted infrastructure and digital resource investments to prevent the “latecomer advantage” from diminishing further.

In terms of regional disparities contribution rates in Fig. 8c, inter-regional disparities are identified as the most important factor, with an average contribution rate of 59.986%. In contrast, intra-regional disparities contribute only 26.615%, significantly lower than inter-regional disparities. This indicates that cross-regional disparities are more pronounced than intra-regional differences. Moreover, hypervariable density reflects the impact of inter-regional interactions on overall disparities with an average contribution of 13.399%. It can be mitigated by strengthening inter-regional innovation exchanges and cooperation to boost the optimal allocation of innovation resources.

## Dynamic evolution of the coupling coordination

Having established the static spatial landscape, it is imperative to investigate the temporal trajectory and state transition mechanisms to understand the system’s sustainability. This section explores the dynamic evolution laws to reveal how the distribution shape morphs over time and to estimate the probabilistic pathways of regional advancement, providing a predictive view of future development trends.

### Kernel density estimation

To intuitively visualize the continuous evolution of regional disparities, Kernel density estimation is employed to fit the probability density curves of the data. This non-parametric approach reveals critical distributional characteristics—such as the number of peaks and the breadth of the curve—thereby diagnosing whether the regions are converging toward a unified equilibrium or diverging into polarized clubs.

Kernel density estimation can identify actual distribution characteristics without relying on prior assumptions or predefined distributions. The wave’s breadth and height indicate the degree of disparities, and the curve’s position indicates the dynamic evolution tendency. A wider and taller wave crest suggests that many provinces are clustered at a particular level of coordinated development, indicating a smaller difference and higher concentration. Conversely, the more dispersed distribution implies greater differences, and the degree of polarization is depicted by the number of peaks. For instance, multiple peaks imply that there are multiple levels of coordinated development or obvious differentiation.

6 $$f(u)=\frac{1}{{nh}}\sum\limits_{{i=1}}^{n} {K\left( {\frac{{{u_i} - u}}{h}} \right)}$$

where *n* is the sample size, *h* is the bandwidth, and $K( \cdot )$ is the Gaussian kernel function.

To examine how the initial coupling coordination degree influences subsequent periods amid dynamic evolution, the joint kernel density method is introduced.

7 $$f(x,y)=\frac{1}{{n{h_x}{h_y}}}\sum\limits_{{i=1}}^{n} {{K_x}(\frac{{{X_i} - x}}{{{h_x}}})} {K_y}(\frac{{{Y_i} - y}}{{{h_y}}})$$

where *n* is the number of observed data, ${h_x}$ and ${h_y}$ are the x-direction and y-direction bandwidths, ${K_x}( \cdot )$ and ${K_y}( \cdot )$ are the corresponding kernel functions.

#### Univariate kernel density

**Fig. 9:**
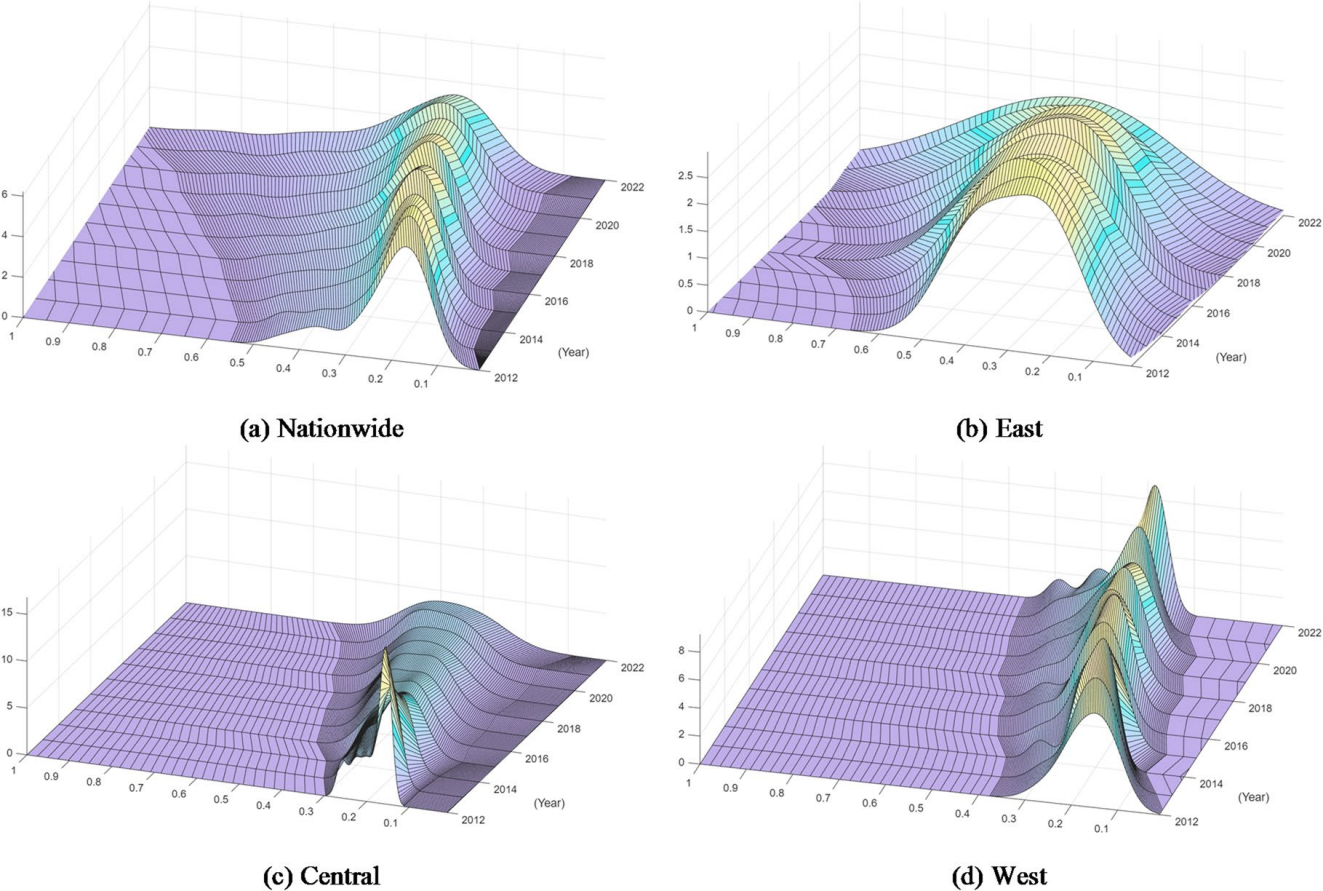
Kernel density curves for coordinated development degree.

This analysis focuses on the temporal progression of the coordination degree’s distribution shape to capture its marginal changes. By observing the shift of the peak and changes in kurtosis over the years, we can assess the overall growth momentum and determine whether the spatial gap is widening or narrowing during the development process. As shown in Fig. 9, it reveals the distribution characteristics of the coupled coordinated degree, and analyzes the characteristics of the evolution trend.

At the national level in Fig. 9a, the evolution curve presents a distinct pattern of “peak lowering and width broadening” accompanied by a rightward shift. This confirms that while the coupling coordination degree is improving overall—moving from the early to mid-stage—the spatial dispersion is increasing. The flattening main peak suggests that the “club convergence” effect is weakening, and the absolute disparity among provinces is widening as more advanced provinces pull ahead.

The three regions still exhibit a striking imbalance in their coupling coordination. The eastern region (Fig. 9b) shows the most significant rightward shift with a broad and flattened distribution, indicating a balanced progression toward high-quality coordination. Its abundant innovation resources have facilitated a comprehensive transformation of productive forces across the region. In contrast, the central region (Fig. 9c) displays a sharp, steep peak that rapidly shifts rightward, reflecting a strong catch-up effect driven by industrial transfer. However, a bimodal tendency emerges in later years, revealing an intensifying internal differentiation where leading provinces are detaching from the average. The western region (Fig. 9d) is characterized by a highly tall and thin peak clustered at the low-value range with a slow rightward movement. The evidence suggests a “low-level trap” or high concentration: the vast majority of western provinces are locked in a low-coordination stage due to historical and geographical constraints. The long right tail indicates that only a few growth poles are emerging, while the overall synergistic development significantly lags behind. Therefore, the western region prioritizes breaking this low-level equilibrium through targeted policy support to leverage local resource endowments.

#### Joint kernel density

To examine the temporal inertia and dynamic correlation of the system, Joint Kernel density estimation plots the relationship between the coordination status at time $\:t$ and $\:t+1$. This visualization helps identify the stickiness of development states, revealing whether regions tend to solidify their positions or exhibit high mobility over consecutive years.

**Fig. 10:**
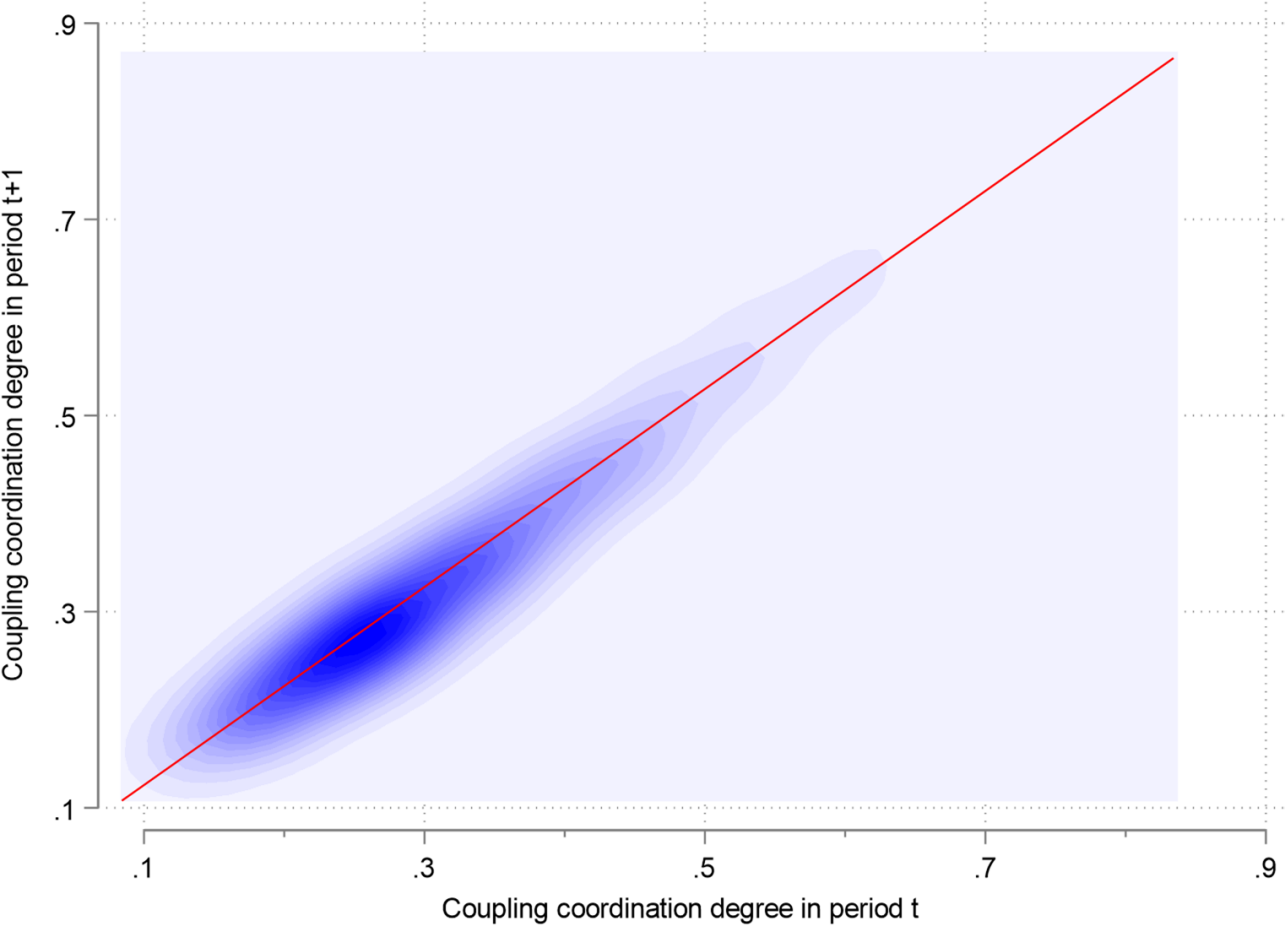
Joint kernel density contours for the coordinated development degree.

In Fig. 10, a contour map is adopted to examine how the coupled coordination degree varies in the future. This map reveals the position of the wave peaks of the coupled coordination degree, along with the tendency to deviate from the diagonal line and shift upward at the national level. At the low coupling coordination development stage, the wave peaks are predominantly located along the diagonal. This indicates a high likelihood of persistence in the subsequent period. In contrast, at the high coupling coordination development stage, the wave peaks are shifted upward from the diagonal. This indicates that the region is inclined to progress to an even higher level in the next period. It reflects the positive potential benefits of regions with higher coupled coordination, such as continuous development, innovation-driven growth, and policy environment.

Regions with high coupling coordination levels have developed self-reinforcing economic mechanisms. Their optimized innovation ecosystems efficiently allocate resources to high-growth industries such as advanced manufacturing, information technology, and biotechnology. Policymakers should formulate strategies to facilitate the spillover of knowledge and technology from high coupling regions to lower ones, thereby fostering more balanced economic growth across the country.

### Markov chain analysis

While density curves illustrate morphological changes, they fail to quantify the specific probability of state transitions. To address this, this study employs the Markov chain method to construct a transition probability matrix. Specifically, the coupling coordination degree is divided into four quartiles, allowing for the calculation of the precise likelihood of regions maintaining their status or leaping to a higher tier. This process is crucial for evaluating the difficulty of breaking development bottlenecks and predicting long-term steady states. Furthermore, previous research has demonstrated that spatial spillover effect from geographical proximity significantly influence regional development evolution^40,41^. The spatial Markov chain analysis framework is constructed by introducing spatial lag. A spatial weight matrix is constructed based on the geographical proximity. The state transition probabilities in each region are modified in accordance with the spatial lag model to elucidate how spatial spillover effects shape the regional development trajectories.

#### Traditional Markov chain

This subsection calculates state transition probabilities by treating regions as independent entities to establish a baseline for mobility analysis. It aims to uncover the club convergence phenomenon, determining the likelihood of regions maintaining their current status and identifying specific equilibrium states where development levels tend to solidify. As is in Table 4, the coupling coordination degree is classified into four discrete levels: low, medium, high, and extreme level (denoted as 1, 2, 3, and 4 respectively).

First, the diagonal factors in the Markov transition matrix exhibit substantially greater probability values compared to their off-diagonal counterparts. This indicates a strong tendency for the coupling coordination development type to remain in its current state rather than transition to a new one. The is due to the high costs associated with transitions, such as technological adoption costs, institutional rigidities, and labor skill shortages. For instance, provinces with low coupling coordination face substantial R&D infrastructure investment burdens to upgrade.

Second, the club convergence phenomenon is evident in the coordinated development evolution. Provinces with high coupling coordination demonstrate a strong tendency to maintain their developmental status in subsequent periods, with rates reaching up to 100% and 81.08% respectively. This is attributed to their self-reinforcing innovation ecosystems, characterized by knowledge spillovers, efficient capital markets, and abundant talent pools. High-tech enterprises in these areas actively foster innovation and collaboration, further consolidating their leading positions. To promote coordinated inter-regional development and narrow the development gap, it is crucial to implement differentiated policies tailored to regions at different development level. Such policies can assist less-developed regions in constructing more robust innovation ecosystems, thereby accelerating their catch-up process and improving overall development balance.

Third, the probability of jump transfer in the type of coupled coordinated development is relatively low, with transfer probabilities across different states not exceeding 27%. Once the region has developed in a given pattern, it is hard to make major short-term deviations. For regions with industry-based productive forces mode, it is challenging to quickly reallocate innovation resources to new industries. Therefore, attention should be paid to short-term effectiveness, as well as long-term investment in promoting the synergistic development.

**Table 4:** Traditional Markov transfer probability matrices.

t/t + 1	*n*	1	2	3	4
1	83	0.7470	0.2530	0.0000	0.0000
2	76	0.0000	0.7368	0.2632	0.0000
3	74	0.0000	0.0135	0.8108	0.1757
4	67	0.0000	0.0000	0.0000	1.0000

#### Spatial Markov chain

Recognizing that regional development is externally conditioned by the geographic environment, the spatial Markov chain incorporates a spatial lag term into the transition matrix. This analysis investigates how the development level of neighboring provinces impacts a region’s upward mobility, effectively testing for the existence of “spatial poverty traps” or “positive spillover premiums” driven by geographical proximity. A spatial Markov transfer probability matrix is used to examine the impact of geographical neighborhood settings on the type transfer, as shown in Table 5.

First, regional economic development is significantly influenced by geographical context. Provinces in economically developed clusters benefit from knowledge spillovers, shared infrastructure, and larger labor markets, reducing innovation costs and enhancing productive forces. For example, in the Yangtze River Delta, close economic cooperation enables seamless knowledge sharing among enterprises, and the well-developed infrastructure supports efficient resource allocation. Conversely, isolated regions face higher innovation costs and lower chances of achieving high coupling coordination. The geographical background significantly influences the transition dynamics, with transfer probabilities varying significantly under different neighborhood characteristics.

Second, the coupling coordinated development of each province exhibits robust regional synergy, leading to the formation of distinct economic communities based on coupling coordination levels. When the neighboring regions are in low coupling coordination state, relatively more provinces exhibit low-level development. Low-coupling regions are dominated by traditional industries with limited inter-regional cooperation and inefficient resource allocation. Conversely, when neighboring regions exhibit the high coupling coordination degree, provinces with similar high coordination tend to cluster, demonstrating a positive incentive effect. High-coupling regions cluster high-tech industries, facilitating the flow of advanced technology, skilled labor, and capital.

Third, influenced by the spillover effects of neighboring regions, the type transition tends to exhibit diffusion effect. When the neighboring area is a high coupling coordination region, the province may enhance its own coordination level through technology diffusion, capital flow, and policy imitation, thereby increasing the probability of shifting to a higher level. However, when the neighboring area is a low-level region, the negative spillover effect is not significant. For example, with high coupled coordination degree neighbors, the probability of shifting to a high-level coupled coordination type also increases (e.g., ${P_{12|3}}(0.3333)>{P_{12}}(0.2530)$). Policies should encourage cross-regional cooperation and facilitate technology diffusion from high-level to low-level regions.

**Table 5:** Spatial Markov transfer probability matrix.

Neighborhood types	t/t + 1	*n*	1	2	3	4
1	1	49	0.8367	0.1633	0.0000	0.0000
	2	8	0.0000	0.7500	0.2500	0.0000
	3	2	0.0000	0.0000	0.5000	0.5000
	4	5	0.0000	0.0000	0.0000	1.0000
2	1	20	0.6500	0.3500	0.0000	0.0000
	2	27	0.0000	0.7778	0.2222	0.0000
	3	10	0.0000	0.0000	0.8000	0.2000
	4	8	0.0000	0.0000	0.0000	1.0000
3	1	9	0.5556	0.4444	0.0000	0.0000
	2	31	0.0000	0.7419	0.2581	0.0000
	3	36	0.0000	0.0278	0.8611	0.1111
	4	24	0.0000	0.0000	0.0000	1.0000
4	1	5	0.6000	0.4000	0.0000	0.0000
	2	10	0.0000	0.6000	0.4000	0.0000
	3	26	0.0000	0.0000	0.7692	0.2308
	4	30	0.0000	0.0000	0.0000	1.0000

When considering the geographical neighborhood background, the transfer of the coupling coordinated type undergoes significant changes. It indicates that the evolution of the coupled coordinated development type is influenced by spatial spillover effects. This further validates the significance of the spatial lag effect. Despite assuming independence in the transfer of coupled coordinated development types across provinces, the spatial lag effect is evident in the transitions (${Q_b}$= 157.893, *P* =0.000). To effectively promote the coordinated development, it is vital to make full use of the positive role exerted by spatial spillover effect. This can be achieved by breaking down geospatial barriers, facilitating inter-regional exchanges, and forming a complementary and mutually beneficial development patterns.

## Conclusions and policy recommendations

### Conclusions

This study investigates the sustainable coupling coordination of NQPF and the allocation of innovation resources using Chinese provincial data from 2012 to 2022. An evaluation framework based on dual-tower MLP neural network model is developed to assess coordination levels. Spatial correlation analysis and Gini coefficient decomposition were used to identify sources of spatial disparities, while kernel density estimation and Markov chain analysis revealed dynamic evolutionary features.

First, the MLP neural network algorithm captures high-dimensional nonlinear interactions and synergy premiums that traditional linear models overlook. Our comparative analysis reveals a significant scissors difference in developed eastern regions, where the coupling coordination based on dual-tower MLP exhibits exponential growth after crossing specific resource accumulation thresholds. This empirically verifies that the relationship between innovation resources and NQPF follows the law of increasing marginal returns, confirming the superiority of deep learning methods in identifying complex economic synergies.

Second, significant spatial clustering and structural heterogeneity are observed. Regions with similar coordination levels tend to agglomerate geographically, with interregional differences identified as the primary source of overall spatial inequality. While the eastern region has formed a high-level coordination cluster driven by digital-green integration, the central and western regions face a threshold bottleneck. The discrepancy between the MLP and traditional models is minimal in these less developed regions, indicating that the nonlinear emergence mechanism of NQPF has not yet been fully triggered due to resource scarcity.

Third, the dynamic evolution reveals a trend of overall improvement but intensifying polarization. Kernel density estimates show that while the national average coordination level is rising, the multimodal distribution in the eastern region suggests a widening internal gap. Provinces with strong digital infrastructure and innovation absorptive capacity have established cumulative advantages, creating a Matthew effect. In contrast, less developed regions relying on traditional resource inputs face increasing risks of marginalization in the new quality era.

Fourth, spatial spillover effects highlight the necessity of cross-regional integration. The study confirms that NQPF development in one region significantly influences its neighbors. However, current spillovers are constrained by administrative barriers and misallocation resource flows, hindering the formation of a unified national market for innovation factors.

### Policy recommendations

First, China should implement a differentiated “threshold-based” allocation strategy tailored to the distinct development stages revealed by the model. For the eastern region, which has already triggered the nonlinear synergy effect, policies should focus on “0-to-1” disruptive innovations by establishing “future industry incubation zones” targeting quantum computing, brain-computer interfaces, and generative AI, thereby maximizing the synergy premium of high-density innovation resources. Conversely, for the central and western regions that are currently in the pre-threshold phase, the priority is to trigger a qualitative leap. Investment should be concentrated on new infrastructure—such as 5G base stations and Industrial Internet nodes—to rapidly cross the critical threshold of digital connectivity required for NQPF, rather than blindly pursuing high-end R&D projects that lack the necessary industrial foundations.

Second, to address the misallocation between resource endowment and innovation needs, the government should operationalize a “computing power for data” exchange mechanism. This involves upgrading the “east data, west computing” strategy from physical infrastructure construction to a factor value trading system. Specifically, the national-level “green computing power trading platform” should be established, allowing the western region to leverage its renewable energy advantages to provide low-cost, low-carbon computing power. In return, the eastern region should offer high-quality industrial data datasets and algorithmic application scenarios. This mechanism converts the western region’s energy advantage and the eastern region’s data advantage into a mutually reinforcing economic cycle, effectively solving the dilemma of “data without computing power” in the east and “electricity without industry” in the west.

Third, policies should foster scenario-driven “innovation consortiums” across administrative boundaries to overcome barriers hindering spatial spillovers. Shifting from simple industrial transfer to “scenario opening”, the government should encourage manufacturing hubs in the central region to open their production lines as “training grounds” or “testbeds” for digital solutions developed in the east. By establishing cross-provincial “chain-master” innovation consortiums, the digital technologies of the east can be deeply integrated with the manufacturing scenarios of the central region, accelerating the transformation of traditional industries into NQPF.

Finally, governance should transition from static evaluation to intelligent monitoring by establishing an AI-driven dynamic monitoring and warning system. Reflecting the methodological contribution of this study, a dynamic monitoring platform should be deployed to real-time track the coupling coordination degree of each province. If the system detects a “coupling fault” where innovation input increases but coordination stagnates, it should trigger an automatic alert to prompt a precise audit of resource misallocation or structural friction, thereby ensuring the long-term sustainability of NQPF development.

### Limitations and future research

While this study illuminates the coordination between NQPF and innovation resource allocation, certain limitations warrant careful consideration to properly contextualize the findings. First, the employment of a time-invariant spatial weight matrix, designed to mitigate endogeneity, inherently assumes structural stability and may therefore understate dynamic spillover effects arising from rapidly emerging regional economies. Second, the reliance on macro-statistical indicators may not fully capture the granular impacts of nascent frontier technologies, such as generative AI, within the evolving NQPF paradigm. Finally, as this research prioritizes the identification of spatiotemporal patterns over strict causal inference, the findings should be interpreted as diagnostic correlations rather than deterministic policy prescriptions, leaving room for future studies to advance the field through dynamic weighting, micro-level data, and robust causal identification strategies.

## Data Availability

The basic data used in the research can be found on the website of National Bureau of Statistics (https://www.stats.gov.cn), China Stock Market & Accounting Research Database (https://data.csmar.com), the International Federation of Robotics (https://ifr.org), Tianyancha (https://www.tianyancha.com), and the Ministry of Industry and Information Technology (https://www.miit.gov.cn).
